# *Chlamydia* infection depends on a functional MDM2-p53 axis

**DOI:** 10.1038/ncomms6201

**Published:** 2014-11-13

**Authors:** Erik González, Marion Rother, Markus C. Kerr, Munir A. Al-Zeer, Mohammad Abu-Lubad, Mirjana Kessler, Volker Brinkmann, Alexander Loewer, Thomas F. Meyer

**Affiliations:** 1 Department of Molecular Biology, Max Planck Institute for Infection Biology, Charitéplatz 1, 10117 Berlin, Germany; 2 Steinbeis Innovation Center for Systems Biomedicine, Falkensee, Germany; 3 Institute for Molecular Bioscience, University of Queensland, St Lucia, Queensland 4072, Australia; 4 Berlin Institute for Medical Systems Biology, Max-Delbrück-Center for Molecular Medicine, Robert-Rössle-Strasse 10, 13125 Berlin, Germany

## Abstract

*Chlamydia*, a major human bacterial pathogen, assumes effective strategies to protect infected cells against death-inducing stimuli, thereby ensuring completion of its developmental cycle. Paired with its capacity to cause extensive host DNA damage, this poses a potential risk of malignant transformation, consistent with circumstantial epidemiological evidence. Here we reveal a dramatic depletion of p53, a tumor suppressor deregulated in many cancers, during *Chlamydia* infection. Using biochemical approaches and live imaging of individual cells, we demonstrate that p53 diminution requires phosphorylation of Murine Double Minute 2 (MDM2; a ubiquitin ligase) and subsequent interaction of phospho-MDM2 with p53 before induced proteasomal degradation. Strikingly, inhibition of the p53–MDM2 interaction is sufficient to disrupt intracellular development of *Chlamydia* and interferes with the pathogen’s anti-apoptotic effect on host cells. This highlights the dependency of the pathogen on a functional MDM2-p53 axis and lends support to a potentially pro-carcinogenic effect of chlamydial infection.

## Abstract (web summary)

Protein p53 plays key roles in cellular stress responses and is frequently deregulated in cancer. Here the authors show that infection with chlamydiae activates the ubiquitin ligase MDM2 in infected cells, leading to proteasomal degradation of p53 and thus promoting apoptosis resistance.

## Graphical abstract

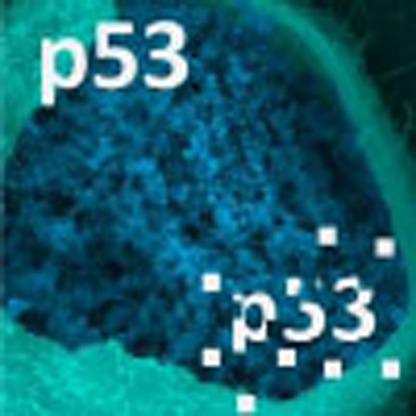

The tumour suppressor p53, the ‘guardian of the genome’, is modulated in response to cellular stress, including DNA damage, osmotic shock, ribonucleotide depletion, deregulated oncogene expression and also by specific pathogenic bacteria^1^,^2^. Activation of p53 initiates a suite of signalling cascades that lead to transient cellular responses (for example, cell cycle arrest and DNA repair) or to terminal cell fates (for example, differentiation, apoptosis or senescence), depending on the nature and degree of the signal initiated^3^.

*Chlamydia trachomatis* is the most common cause of sexually transmitted bacterial infection in humans and the leading cause of preventable blindness worldwide^4^. If left untreated, infection in women can lead to pelvic inflammatory disease, causing chronic pelvic pain and even infertility. In addition, positive correlations between *C. trachomatis* infection and invasive cervical cancer (ICC) in human papillomavirus (HPV)-positive women suggest that *Chlamydia* can act as co-factor for squamous cell transformation^5^,^6^,^7^, although the molecular mechanism for this correlation is unclear.

Depending on the infection stage, *Chlamydia* can induce host cell death or actively inhibit apoptosis^8^. Initial observations demonstrated a profound resistance of *C. trachomatis*-infected HeLa cells to a range of apoptotic stimuli^9^, a property that was shared with other pathogenic *Chlamydia* species^10^. Our recent finding that cells cleared of *Chlamydia* infection exhibit reduced p53 binding to the promoter of the cell cycle checkpoint regulator p21 (ref. 11), together with the central role of p53 in modulating the cellular stress response, notably apoptosis, prompted us to investigate the consequences of *Chlamydia* infection on cellular p53 levels.

Here we find that p53 is proteolytically degraded from 24 hours post-infection (h p.i.) with various *Chlamydia* species in response to activation of the classical p53–MDM2 interaction axis. Further, we find that pharmacological inhibition of this interaction is sufficient to inhibit both the intracellular development of the pathogen and re-sensitize the infected host cells to apoptotic stimuli.

## Results

### *Chlamydia* infection induces the degradation of p53

**Figure 1:**
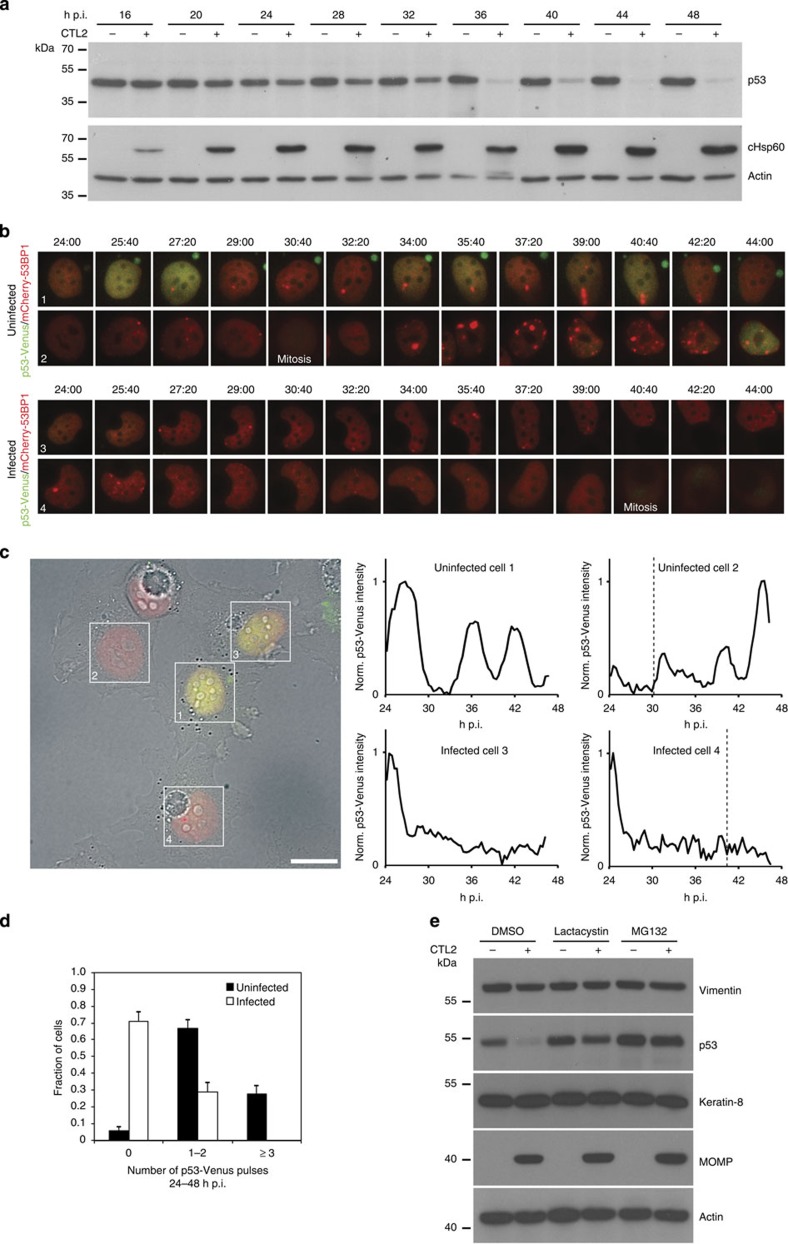
*Chlamydia* induces p53 degradation. (**a**) Western blotting analysis showing progressive degradation of total p53 protein between 24 and 48 h p.i. in CTL2*-*infected whole-cell lysates (MOI=1). Chlamydial Hsp60 and β-actin served as infection and loading controls, respectively. (**b**) Representative time-lapse microscopy images of p53-Venus-expressing cells infected with CTL2 (MOI=0.5) from 24 to 40 h p.i. Nuclei from infected and uninfected cells are presented as montages as indicated. (**c**) Individual cells were tracked and the average nuclear Venus fluorescence (in green) measured. Normalized trajectories of p53-Venus levels are shown. Vertical dashed line indicates time of cell division. Scale bar, 20 μm. (**d**) The fraction of cells showing no pulse, 1 or 2 pulses or more than 3 pulses between 24 and 48 h p.i. The frequency of p53 pulses from >60 infected or uninfected cells were quantified. Error bars present the standard error of the proportion. (**e**) Western blotting showing decreased degradation of total p53 and non-cleavage of keratin 8 and vimentin in whole-cell lysates prepared from cells infected with CTL2 (MOI=1) for 48 h and treated with the proteasome inhibitors lactacystin or MG132 (150 μM) from 47 h p.i. Chlamydial Major Outer Membrane Protein (MOMP) and β-actin served as infection and loading controls, respectively. Full blots for **a** and **e** are shown in Supplementary Fig. 7.

Western immunoblotting of HeLa cells infected with *Chlamydia trachomatis* L2 (CTL2) revealed a striking reduction in total cellular p53 protein starting from 24 h p.i., with a further reduction to nearly non-detectable levels between 36 and 48 h p.i. (Fig. 1a). The *Chlamydia*-induced p53 degradation was observed irrespective of the cell lysis and protein sample preparation procedure applied (Laemmli, 8 M urea or RIPA buffer; Supplementary Fig. 1a,b), excluding proteolytic artefacts^12^. Also, the degraded protein was confirmed to be tumour-suppressor p53 using specific RNA interference oligonucleotides (Supplementary Fig. 1c).

Previous studies have demonstrated that population-averaged analyses such as immunoblotting can mask true dynamic signalling responses to stress^13^,^14^. Furthermore, we sought to circumvent the necessary lysis steps inherent in these protocols as there is currently significant controversy surrounding the actions of chlamydial proteases following cell lysis^12^. We therefore analysed p53 dynamics in intact cells using an MCF7 reporter cell line expressing pMT-p53-Venus and pEF1α-mCherry-53BP1. These cells present spontaneous asynchronous pulses in the fluorescent p53-Venus signal over time^15^. Cells were infected with CTL2 and examined by time-lapse videomicroscopy from 24 to 48 h p.i. Strikingly, although p53-Venus oscillations (p53 pulses) were observed in uninfected cells, infected cells (identified by their large *Chlamydia*-filled inclusions) presented a rapid and sustained reduction of p53-Venus signal from 24 to 48 h p.i., concurrent with an increase in inclusion size (Fig. 1b–d; Supplementary Movie 1). As the expression of this p53-Venus reporter is under the control of a constitutive metallothionein (MT) promoter, this observation suggests that the decrease in cellular p53 observed in Fig. 1a must reflect proteolytic activity rather than transcriptional regulation.

**Figure 2:**
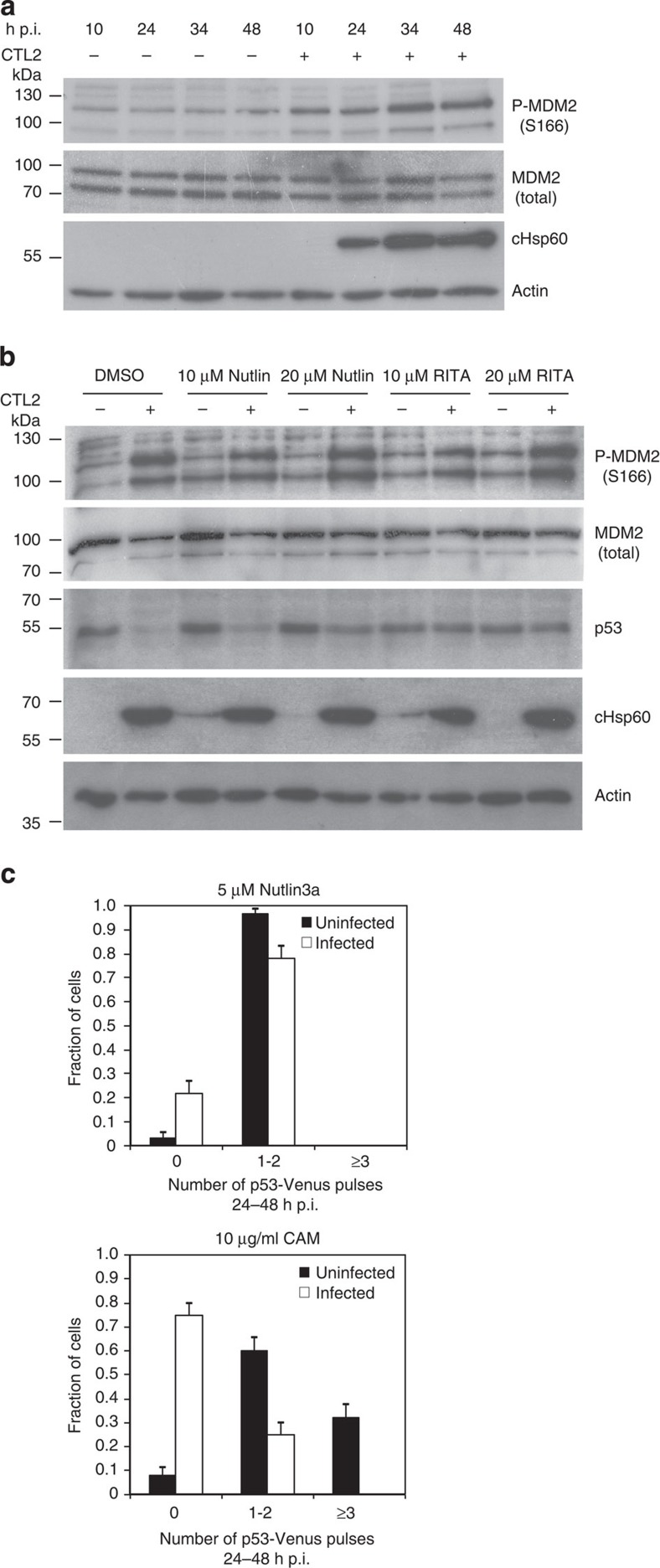
*Chlamydia*-induced degradation of p53 is mediated by phosphorylation of MDM2. (**a**) Western blotting analysis showing a progressive increase of P-MDM2 (S166) compared with total MDM2 between 10 and 48 h p.i. in CTL2 (MOI=1)-infected whole-cell lysates. Chlamydial Hsp60 and β-actin served as infection and loading controls, respectively. (**b**) Western blotting showing decreased degradation of total p53 in whole-cell lysates prepared from cells infected with CTL2 (MOI 1) for 48 h and treated with the inhibitors Nutlin3a or RITA. Chlamydial Hsp60 and β-actin served as infection and loading controls, respectively. Full blots for **a** and **b**) are shown in Supplementary Fig. 7. (**c**) The fraction of cells showing no pulse, 1 or 2 pulses or more than 3 pulses between 24 and 48 h p.i. was quantified following treatment with Nutlin3a or chloramphenicol (CAM). The frequency of p53 pulses from >60 infected or uninfected cells was quantified. Error bars present the standard error of the proportion.

Regulation of p53 degradation is mediated by Murine Double Minute 2 (MDM2). Following activation, MDM2 binds and ubiquitinates p53, leading to its translocation to the cytosol and proteasomal degradation^16^. MDM2 is phosphorylated by AKT at Ser-166/186, both of which are located within the RXRXXS/T consensus motifs for AKT kinase. The phosphorylation of MDM2 at Ser-166/186 promotes its nuclear localization and its interaction with p300, a transcriptional co-activator that forms a complex with MDM2 and promotes p53 degradation^17^,^18^,^19^. To monitor MDM2 activation, we carried out western immunoblotting of *Chlamydia*-infected protein samples using an anti-phospho-MDM2 (Ser166) antibody. From 10 h p.i. an elevation in phosphorylated MDM2 was observed, suggesting that this might be an essential step involved in p53 degradation in *Chlamydia*-infected cells (Fig. 2a). Notably, western immunoblotting of the same samples using antibodies to detect total MDM2 levels showed no change following infection. Inhibiting proteasomal activity with the cell permeable proteasome inhibitors MG132 and lactacystin^20^–also a known inhibitor of the chlamydial protease-like activity factor (CPAF)–rescued p53 protein levels (Fig. 1e), indicating the involvement of the proteasome in *Chlamydia*-induced p53 degradation. To exclude artificial post-lysis degradation of p53 by CPAF, we also monitored cleavage of keratin-8 and vimentin, two recognized post-lysis CPAF targets^12^,^21^. No evidence of keratin-8 or vimentin proteolysis was observed at 48 h p.i. (Fig. 1e) thus validating our lysis method.

To dissect the link between *Chlamydia*-induced p53 degradation and MDM2 phosphorylation, we used established pharmacological inhibitors as they offer the capacity to rapidly and specifically target key molecular players. Nutlins are *cis-*imidazoline analogues that inhibit the interaction between phospho-MDM2 and p53 (ref. 22). Similarly, the small-molecule inhibitor RITA (reactivation of p53 and induction of tumour cell apoptosis) binds p53 and thereby also prevents its interaction with MDM2 and the subsequent degradation of p53 (ref. 23). Cells were infected with CTL2 for 48 h, treated with Nutlin3a or RITA at the indicated concentrations from 12 h p.i., and subjected to western immunoblotting. The inhibitors markedly reduced the degree to which p53 was degraded, whereas levels of phosphorylated MDM2, total MDM2 and bacterial Hsp60 protein remained unchanged (Fig. 2b). Further, time-lapse videomicroscopy demonstrated that 5 μM Nutlin3a added at 24 h p.i. was sufficient to rescue the pulses in p53-Venus, despite infection with CTL2 (Fig. 2c). Interestingly, although infected cells presented regular pulses in p53-Venus signal with Nutlin3a treatment, uninfected cells presented a sustained p53-Venus signal (Supplementary Movie 2). Treatment with the bacterial protein synthesis inhibitor chloramphenicol (CAM, 10–100 μg ml^−1^) from 24 h p.i. was sufficient to inhibit inclusion expansion^24^, however, it was not sufficient to rescue the p53-Venus pulses (Fig. 2c and Supplementary Movie 3) or prevent phosphorylation of MDM2 and degradation of p53 (Supplementary Fig. 2).

There is considerable controversy surrounding the roles played by secreted bacterial proteases during chlamydial infection and the associated protein degradation phenotypes observed^25^. Treatment of *Chlamydia*-infected cells with CAM is sufficient to reverse a number of cellular phenotypes induced upon infection^26^,^27^,^28^, presumably reflecting an inability to maintain sufficient intracellular concentrations of secreted bacterial effector molecules following application of the agent. Indeed, time-lapse videomicroscopy of *Chlamydia-*infected cells treated with 10 μg ml^−1^ CAM from 24 h p.i. and imaged from 48 to 88 h p.i. continued to present a lack of p53-Venus pulses (Supplementary Movie 4). To examine the possible role played by CPAF in this process, a published cell permeable CPAF-specific inhibitor peptide^29^ was employed in conjunction with time-lapse videomicroscopy. As reported previously, addition of the CPAF-inhibitor peptide from 12 or 24 h p.i. led to rapid induction of non-apoptotic cell death in CTL2-infected cells, severely attenuating the duration that videomicroscopy could be performed^29^. In spite of this, no p53 pulses were observed from 24–40 h p.i. in *Chlamydia-*infected cells cultured in the presence of the inhibitor peptide, whereas p53 pulses were still readily observed in uninfected cells (Supplementary Movie 5).

### *Chlamydia* requires functional interaction of MDM2 with p53

**Figure 3:**
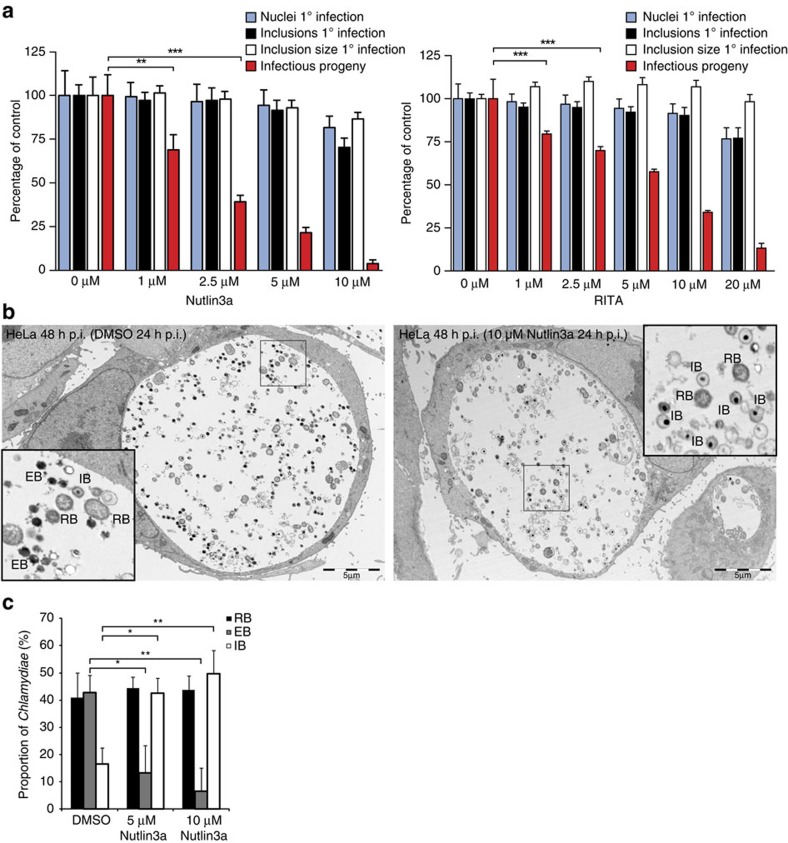
Disruption of p53–MDM2 interaction inhibits the formation of infectious progeny. (**a**) Monolayers of HeLa cells, infected with CTL2 (MOI 0.5) for 24 h p.i., and treated with or without Nutlin3a or RITA for additional 24 h, were labelled with anti*-Chlamydia* antibody and Hoechst and the number and size of inclusions as well as number of nuclei per well determined using Scan^R^ software. From parallel wells, the generation of infectious EBs was determined via infectivity assay. Results depicted as mean percentage±s.d. normalized to controls of two independent experiments; **P<0.01, ***P<0.001, one-way analysis of variance with Bonferroni *post-hoc* test. (**b**) Representative transmission electron micrographs of infected cells 48 h p.i., with or without Nutlin3a from 24 h p.i. Scale bars, 5 μm. Insets present high-resolution images of the indicated region. (**c**) The relative proportion of EBs, RBs and IBs, quantified from 5 to 6 electron micrographs of three independent experiments. Nutlin3a treatment significantly increased the proportion of IBs, whereas the proportion of EB’s decreased accordingly. Results are depicted as mean percentage±s.d. of two independent experiments; *P<0.05, **P<0.01, two-tailed Student’s *t*-test.

A moderate reduction in the expansion of inclusions in infected cells treated with 5 μM Nutlin3a was noted in the time-lapse experiments (compare Supplementary Movies 1 and 2). This prompted us to investigate the consequences of Nutlin3a treatment on the formation of infectious CTL2 progeny. Cells were infected with CTL2 for 24 h to establish infection and then treated with increasing concentrations of Nutlin3a or RITA for a further 24 h. The cells were then lysed and the lysates used to infect a second population of HeLa cells for 24 h. Cells were fixed, immunolabelled and the number of resulting inclusions quantified. In parallel, the impact of Nutlin3a or RITA on inclusion size and number as well as the number of host cell nuclei was monitored in the primary infection. Strikingly, although there was little significant impact upon inclusion size or number in the primary infection following Nutlin3a or RITA treatment, there was a dramatic, dose-dependent decrease in infectious progeny, suggesting that the pathogen was unable to complete its normal developmental cycle (Fig. 3a).

Chlamydial development is characterized by the reversible transition from an electron-dense elementary body (EB) to a larger, non-infectious replicative reticulate body (RB). During the later stages of the infection cycle, the replicating RBs transform back into infectious EBs before host cell lysis or extrusion of the inclusion. Ultrastructural analysis by transmission electron microscopy of HeLa cells infected for 48 h with CTL2 in the presence or absence of 5 or 10 μM Nutlin3a from 24 h p.i. revealed that Nutlin3a did not induce major morphological or structural changes in inclusion or bacterial membranes, nor were enlarged RBs, often referred to as aberrant bodies, observed (Fig. 3b). However, there was a highly significant reduction in the proportion of EBs coupled with a concurrent increase in the proportion of intermediate bodies (IBs)–RBs in the process of retransformation to EBs–characterized by their size and compact nucleoids (Fig. 3b,c). Similar observations were made in RITA-treated cells (Supplementary Fig. 3). This suggests that a functional MDM2–p53 interaction is necessary for the proper formation of infectious EBs.

### Degradation of p53 is conserved among pathogenic *Chlamydiae*

**Figure 4:**
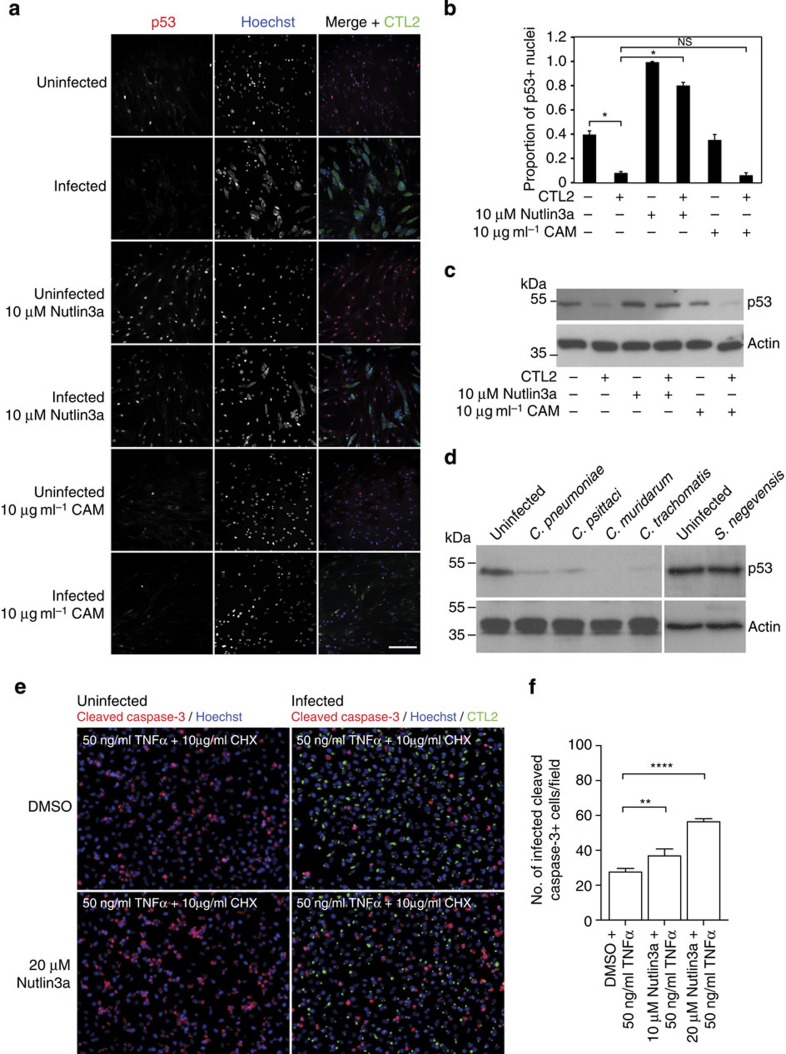
Degradation of p53 is sustained in primary cells and mediates *Chlamydia*’s anti-apoptotic effect. (**a**) CTL2 infection reduces the number of p53-positive nuclei in fallopian tube primary mesenchymal cell monolayers, an effect which is partially reversed by Nutlin3a treatment. Chloramphenicol (CAM) has no effect on p53 degradation in infected cells. Primary cells were infected with CTL2 (MOI 1) and treated with Nutlin3a or CAM from 24 to 48 h p.i. *Chlamydia* inclusions and p53 were labelled with a *Chlamydia* and p53 antibody, respectively; nuclei were stained with Hoechst. Scale bar, 200 μm. (**b**) Quantification of the confocal images shows that the Nutlin3a-induced increase in the proportion of p53-positive nuclei is highly significant. Results are depicted as mean±s.d. of two independent experiments; *P<0.05, two-tailed Student’s *t*-test. (**c**) Western blotting showing decreased degradation of total p53 in whole-cell lysates prepared from human fallopian tube primary cells infected with CTL2 (MOI=1) for 48 h. Nutlin3a treatment recovered p53 protein levels, whereas CAM treatment did not; β-actin served as loading control. (**d**) Western blotting analysis showing that in human fallopian tube primary cells infected with *C. pneumoniae* (MOI=5), *C. psittaci* (MOI=3), *C. muridarum* (MOI=1) or *C. trachomatis* L2 (MOI=1) p53 protein levels are dramatically reduced by 48 h p.i. compared with uninfected cells, whereas total p53 protein 80 h p.i. in *Simkania negevensis-*infected (MOI=2) cells is comparable to controls; β-actin served as loading control. Full blots for **c** and **d** are shown in Supplementary Fig. 7. (**e**) The extent of apoptosis induced by TNF-α treatment was analysed by immunolabelling with cleaved caspase-3 antibody in HeLa cells infected with CTL2 (MOI=1). Infected cells showed greatly reduced labelling, however, Nutlin3a treatment partially restored the number of cleaved caspase-3-positive cells. *Chlamydia* inclusions and nuclei were labelled with *Chlamydia* antibody and Hoechst. (**f**) Number of cleaved caspase-3-positive nuclei per image in infected HeLa cells treated with Nutlin3a during apoptosis induction with TNF-α and cycloheximide, shows that Nutlin3a significantly increases the number of apoptotic nuclei in infected cell populations. Results are depicted as mean±s.d. of three independent experiments; **P<0.01, ****P<0.0001, one-way analysis of variance with Bonferroni *post-hoc* test.

The primary site of CTL2 infection is the urogenital system. To examine the impact of CTL2 infection upon p53 in cells of this system, human primary mesenchymal cells were prepared from fallopian tubes surgically removed from patients undergoing procedures for benign gynaecological disease, as described previously^30^. These primary cells were infected with CTL2 at multiplicity of infection (MOI)=1 and treated with Nutlin3a or CAM from 24 to 48 h p.i. The samples were then fixed and prepared for immunofluorescence microscopy using antibodies against p53 and CTL2 (Fig. 4a). Confocal analysis confirmed that while around 40% of non-infected cells presented a detectable p53 signal in the nucleus, this proportion decreased significantly to less than 10% upon infection, in congruence with our observations in HeLa and MCF7 cells. Similarly, the decrease in p53 signal could be rescued following treatment with 10 μM Nutlin3a but not with 10 μg ml^−1^ CAM (Fig. 4b). As in HeLa cells, CTL2 infection did not influence total cellular MDM2 levels of primary fallopian tube mesenchymal cells (Supplementary Fig. 4). Western immunoblotting of primary fallopian tube mesenchymal cells infected with CTL2 and treated with Nutlin3a confirmed that *Chlamydia*-induced p53 degradation was dependent upon a functional p53–MDM2 interaction (Fig. 4c). CAM treatment was not sufficient to prevent degradation of p53 in primary mesenchymal cells from the fallopian tube (Fig. 4c). These observations in primary cells are in accordance with the western immunoblotting and live cell microscopy data of HeLa cells presented in Fig. 2b,c, Supplementary Movies 2 and 3 and Supplementary Fig. 2.

To investigate whether or not this phenotype was species-specific, the impact of infection with *C. pneumoniae*, *C. psittaci, C. muridarum* and *C. trachomatis* L2 on p53 protein levels in primary fallopian tube cells was compared. Western immunoblotting demonstrated that in all cases infection resulted in a significant decrease in p53 (Fig. 4d). In contrast, infection with *Simkania negevensis*^31^,^32^–a primarily environmental bacterial member of the order of *Chlamydiales*–did not induce degradation of p53 (Fig. 4d; Supplementary Fig. 5). This observation suggests that p53 degradation results from a direct impact of the invasive *Chlamydiaceae* upon the host cell rather than from general, unspecific infection-induced stress.

### Loss of p53 contributes to *Chlamydia*’s anti-apoptotic influence

*Chlamydia*-infected cells are profoundly resistant to apoptosis during the replicative phase of the pathogen’s development^8^. To determine whether the observed p53 degradation contributes to this apoptosis resistance, HeLa cells at 12 h p.i. were treated with 20 μM Nutlin3a for 10 h, followed by induction of apoptosis with 50 ng ml^−1^ tumour necrosis factor-α (TNF-α) and 10 μg ml^−1^ cycloheximide for 5 h. Cells were fixed and examined by immunofluorescence microscopy using antibodies directed against cleaved caspase-3. Although *Chlamydia* infection alone conferred significant resistance to TNF-α-induced apoptosis, inhibition of MDM2-mediated degradation of p53 with Nutlin3a was sufficient to partly reverse this phenotype (Fig. 4e,f). An alternative readout of apoptosis was applied by using antibodies directed against cleaved poly-(ADP-ribose) polymerase (PARP). Again, Nutlin3a treatment of infected cells partly re-sensitized cells to the pro-apoptotic signal elicited by TNF-α as monitored by the significant increase in the number of infected and cleaved PARP-positive cells, thus reinforcing our observation that recovery of p53 protein levels through inhibition of the MDM2–p53 interaction impairs the anti-apoptotic effect induced by *Chlamydia* (Supplementary Fig. 6). Indeed, we noted that infected MCF7 p53-Venus reporter cells were also more sensitive to photodamage during the live-cell imaging experiments following treatment with Nutlin3a (Supplementary Movie 2), indicating that they are more sensitive to extracellular stress stimuli, again reinforcing the connection between the degradation of p53 and the resistance to apoptosis observed in *Chlamydia*-infected cells.

Therefore, MDM2-mediated degradation of p53 in *Chlamydia*-infected cells contributes to the anti-apoptotic influence upon the host cell.

## Discussion

In order to complete its unique biphasic developmental cycle, *Chlamydia* has developed potent strategies to protect infected cells against death-inducing stimuli^8^. The metabolic burden placed upon the host cell during the replicative phase and the later stages of chlamydial infection initiates a number of stress-related signalling pathways, which in turn need to be modulated by the pathogen to maintain host cell viability, thereby ensuring the survival of *Chlamydia* and allowing for a productive infection^33^,^34^,^35^. Here we reveal the *Chlamydia*-induced degradation of p53, a key molecular node in the cellular stress response pathway. This degradation was preceded by the activation of the canonical p53-MDM2 axis and could be reversed through the application of specific pharmacological inhibitors of this interaction.

*Chlamydia* infection has recently been shown to cause accumulation of double-stranded DNA breaks within the genome of the host cell while simultaneously interfering with the DNA damage response, notably activation of the key transducer of double-stranded DNA break signals, the nuclear protein kinase ataxia telangiectasia mutated^36^. Thus, the p53 degradation phenotype reported here represents another example of how *Chlamydia* infection interferes with the host’s genotoxic stress response. As the p53 degradation phenotype was observed with all *Chlamydia* species tested, as well as in the presence of the bacterial protein synthesis inhibitor CAM, activation of the MDM2 feedback loop is likely to occur via a host-derived mechanism. One attractive candidate for this role is the AKT/PKB serine-threonine kinase. MDM2 associates with AKT and is phosphorylated at serine residues 166 and 186 following activation of the kinase (through phosphorylation at residues 308 and 473) or ectopic expression of constitutively active AKT. AKT itself is activated in response to a variety of cell survival stimuli via the phosphatidylinositol 3-kinase signalling cascade^37^,^38^. Verbeke *et al.*^39^ demonstrated that inhibition of both phosphatidylinositol 3-kinase activity and AKT expression renders CTL2-infected cells sensitive to staurosporine-induced apoptosis. Further, the authors demonstrated that AKT itself was phosphorylated at serine residue 473 following infection^39^, supporting the consideration that this upstream signalling cascade is instrumental for the degradation phenotype presented here. In addition, however, the inability of Nutlin3a treatment to re-sensitize the infected cells to apoptotic stimuli to the degree seen in uninfected cells strongly suggests that additional mechanisms are employed by the pathogen^40^,^41^,^42^,^43^.

We show that treatment with Nutlin3a and RITA inhibits the later stages of the pathogen’s developmental cycle and thereby blocks the formation of infectious progeny. Precisely, why p53 degradation supports progression of the pathogen’s intracellular development is unclear. Intriguingly, the accumulated IB phenotype observed in Nutlin3a-treated cells is distinctive and remarkably similar to that observed when chlamydial lipooligosaccharide synthesis is inhibited with small molecules, although the agents used in this study share no structural relationship with Nutlin3a^44^. Given p53’s central role in cellular metabolism^45^ and the dependence of the pathogen upon the host for metabolites, metabolomic studies under these conditions may prove to be enlightening.

Finally, given the prominent role of p53 in tumour suppression, one wonders about the contribution of *Chlamydia*-induced p53 degradation to tumourigenesis in the female genital tract. Indeed, a number of epidemiological studies have linked *Chlamydia* infection with several gynaecological cancers, including cervical and ovarian cancers^46^, and carcinogenesis in other organs^47^. It is established that infection with HPV is necessary but not sufficient to induce ICC, as only a small proportion of women infected with HPV progress to develop ICC^48^. This implicates the involvement of additional genetic or environmental factors including infectious agents that may contribute to final transformation. Given that *C. trachomatis* is the most common sexually transmitted bacterium in humans, it represents an attractive candidate for such a co-factor. Several large cohort studies have found a significant correlation between *C. trachomatis* infection and an increased risk of ICC development when adjusted for other factors^5^,^6^,^49^, whereas a single study opposed these epidemiological findings^50^. Thus, *C. trachomatis* infection may play a role in confounding squamous cell ICC in HPV-infected women.

Intriguingly, p53 degradation is an important virulence mechanism in the infection of cervical cells with oncogenic HPV strains 16 and 18 (ref. 51). The E6 and E7 open reading frames of HPV encode oncogenes, of which E7 binds to tumour-suppressor protein pRB leading to its degradation via the ubiquitin-proteasome pathway^52^,^53^ and E6 forms a complex with p53 and E6AP, an E3 ubiquitin ligase, promoting p53 degradation via the same pathway^54^,^55^. Co-infection with *C. trachomatis* might augment the action of the E6 HPV protein by activating MDM2-dependent degradation of p53 and shift cell fate further towards transformation.

Our observations with normal human cells derived from the fallopian tube, which is not a primary site of HPV infection, further suggest that *C. trachomatis* could induce severe p53 degradation even in the absence of E6. It has recently been suggested that a significant proportion of high-grade serous ovarian cancer originates from the columnar epithelium of the fallopian tube^56^,^57^,^58^, which is readily colonized by *C. trachomatis* during ascending infections. Intriguingly, p53 loss of function is a common denominator of nearly all ovarian cancer cases^59^. Functional p53 signalling is likely to be an essential response to the cytotoxic stress triggered in the fallopian tube by ovulation^60^. Chronic or recurrent infections with *C. trachomatis* could interfere with this protective mechanism. Hence, our study also lends support for a possible direct role of *C. trachomatis* infection in ovarian cancer development. Although more comprehensive insight is required to determine the mechanistic contribution of *C. trachomatis* to these malignancies, the observation of p53 degradation may be considered a significant piece of the puzzle.

## Methods

### Antibodies, cell lines, human tissue samples and reagents

Mouse monoclonal anti-p53 (DO-1, sc-126, 1:1,000–2,000 for immunoblotting, 1:100 for immunofluorescence) and rabbit anti-total MDM2 (N-20, sc-813, 1:1,000 for immunoblotting, 1:100 for immunofluorescence) were purchased from Santa Cruz Biotechnology Inc.; anti-*Chlamydia trachomatis* MOMP (ab20881, 1:2,000), anti-cytokeratin-8 (ab9023, 1:8,000) and anti-vimentin (ab16700, 1:2,000) antibodies were purchased from Abcam, mouse monoclonal anti-*Chlamydia trachomatis* species-specific KK-12 IgG2a, was obtained from David Grayston (University of Washington, Seattle, WA, USA, 1:10,000), genus-specific rabbit polyclonal anti-*Chlamydia* (3-090, 1:100) from Milan Analytica, mouse monoclonal anti-*C. trachomatis* Hsp60 (ALX-804-072-R100, 1:8,000) from Enzo Life Sciences; rabbit monoclonal anti-cleaved PARP (Asp214, D64E10, 1:400) from Cell Signaling, mouse monoclonal anti-β-actin (AC-15, 1:4,000–16,000) from Sigma, rabbit polyclonal anti-cleaved caspase-3 (Asp175, 9661, 1:400) and rabbit polyclonal anti-P-MDM2 (S166, 3521S, 1:1,000) were from Cell Signaling. Secondary antibodies used for immunofluorescence and western blot analyses were Cy3-linked anti-rabbit (711-165-152, 1:200) and anti-mouse (115-166-072, 1:200), Cy2-linked anti-mouse (115-225-146, 1:200) and DyLight 488 anti-rabbit (111-485-144, 1:200) from Jackson ImmunoResearch Laboratories, HP-linked ECL anti-rabbit (NA934, 1:3,000) and HP-linked anti-mouse (NA931, 1:3,000) from Amersham. HeLa (ATCC CCL-2) and the p53-Venus reporter MCF7 cell lines^15^ were maintained in RPMI-1640 medium (52400) from Gibco-Life Technologies (52400) supplemented with 10% fetal bovine serum (S0115, Biochrom AG). Nutlin3a was purchased from Sigma (N6287), RITA from Calbiochem (506149), MG132 from Sigma (C2211) and Lactacystin from Cayman Chemical (CAS 154226-60-5). CPAF-inhibitor peptide (SLFYSPMVPHFWAELRNHYATSGLKRRRRRRRRR) and the scrambled control (NFALSHFRLPLSTYKEMPYVSHWAGRRRRRRRRR) with >95% purity were ordered from Genscript. A dose-dependent reduction in infectious progeny and induction of cell death, as reported previously^61^, was used to confirm its efficacy. RNA interference oligonucleotides siAllstars (scrambled control) and sip53 (targeting p53, 5′-TTGGTGAACCTTAGTACCTAA-3′) were purchased from Qiagen. Hoechst 33342 was purchased from Sigma.

Human fallopian tube tissue samples were provided by the Department of Gynaecology, Charité University Hospital, Campus Virchow Clinic, Berlin, Germany. Scientific use of the tissue was approved by the Ethics Commission of the Charité, Berlin (EA1/002/07) and informed consent was obtained from all subjects. Fragments were sourced from standard surgical procedures for benign gynaecological diseases and only anatomically normal pieces were utilized for subsequent experiments. Primary mesenchymal cells were prepared from these samples as described previously^30^ and typically used for infection at passage numbers six to eight.

### Infections with *Chlamydiales*

We used *Chlamydia trachomatis* L2 (ATCC VR-902B), *Chlamydia pneumoniae* (ATCC VR-1310), *Chlamydia muridarum* (ATCC VR-123), *Chlamydia psittaci* (02DC15) and *Simkania negevensis* (ATCC VR-1471) at the indicated MOIs. Cells were infected for 2 h in normal RPMI growth medium containing 5% fetal bovine serum, 2 mM glutamine and 1 mM sodium pyruvate at 35 °C. The medium was then replaced with fresh growth medium and cells were cultured for the indicated times at 35 °C. In addition, *C. pneumoniae* and *C. psittaci* inocula were centrifuged at 920 × *g* for 30 min onto fresh cell monolayers and then further incubated under the conditions described above.

### Western immunoblotting

Cell monolayers were lysed directly with SDS lysis buffer (100 mM Tris/HCl, pH 6.8, 4% SDS, 20% glycerol, 0.02% bromophenol blue, 200 nM dithiothreitol), where indicated with 8 M urea^12^ or RIPA lysis buffer (50 mM Tris/HCl pH 7.5, 1% Nonidet P-40, 0.1% SDS, 150 mM NaCl, 2 mM EDTA). RIPA lysates were incubated for 30 min on ice. Cell lysates were boiled at 95 °C for 10 min. Equal amounts of protein were separated using SDS–polyacrylamide gel electrophoresis and immunoblotted as described previously^62^.

### Time-lapse videomicroscopy

This procedure was conducted on cells seeded onto glass-bottom 35 mm dishes (Mattek) in an environment-controlled (37 °C and 5% CO_2_) system consisting of an Olympus IX81 inverted microscope (Olympus), X-Light Multipoint Confocal Scanning Module (CrEST) with Nipkow spinning disc, and a Hamamatsu C9100-02 CCD camera (Hamamatsu Photonics K.K). Bright-field images were acquired with a × 63 phase contrast objective (NA=1.25 oil, Olympus) and a high-speed shutter system. Fluorescent images were acquired with a Lumencor SPECTRA-4 LED-based Light Engine Illumination System with 390/18; 475/28; 549/15; 632/22 solid-state LEDs using VisiView Premier Image acquisition Software. To examine the impact of the CPAF-specific inhibitor peptide time-lapse videomicroscopy was conducted similarly on a Nikon Ti-E inverted deconvolution microscope equipped with a Hamamatsu 4.0 4Mp Mono camera, × 40/0.95 Apo DIC W.D. 0.25 mm objective, Lumencor Spectra 7 epifluorescent lamp and climate control chamber. In short, the reporter cells were infected for 2 h (MOI=0.5), washed and incubated at 37 °C and 5% CO_2_ for either 12 or 24 h. Media were then replaced with media containing 0, 2.5, 5, 7.5, 10, 12.5, 15, 20, 30 μM CPAF peptide or control peptide. Videomicroscopy was conducted from 24 h p.i. Acute cytotoxicity was observed at CPAF inhibitor peptide concentrations >15 μM. P53-Venus pulses were not observed in infected cells at any of the CPAF-peptide concentrations tested but cytotoxic effects limited recording times to between 10 and 20 h. P53 pulses were still evident in uninfected cells cultured in the presence of the CPAF peptide. The interval between frames was 20 min and exposure times were <50 ms to minimize photobleaching and phototoxic effects. Post-acquisition processing and analysis was conducted using FIJI software.

### Infectious progeny assays

HeLa cells seeded in 384-well plates were infected with CTL2 as described above. Infected cells were lysed at 48 h p.i. by adding Nonidet P40 (NP40) at a final concentration of 0.06% per well. Pipetting steps were performed using a robotic station (Biomek FX pipetting robot, Beckman Coulter). Lysates were diluted in infection medium and were transferred to fresh HeLa cells at a final dilution of 1:40. After 2 h medium was exchanged. Plates were incubated for 24 h at 35 °C and 5% CO_2_ and processed for immunolabelling.

### Transmission electron microscopy

Samples were fixed with 2.5% glutaraldehyde, post-fixed with 0.5% osmiumtetroxide, contrasted with 2% uranylacetate and 0.1% tannic acid, dehydrated with a graded ethanol series and epoxy resin-embedded. Ultrathin sections were contrasted with lead citrate and analysed using a Leo 906E transmission electron microscope.

### Indirect immunofluorescence

This procedure was conducted as described previously^62^. Briefly, cell monolayers were fixed for 25 min in 4% paraformaldehyde followed by liberal washing with PBS. Monolayers were permeabilized for 10 min with Triton X-100 (0.3%) in PBS, washed three times and then incubated with 2.5% BSA in PBS as a blocking reagent. Cells were sequentially incubated for 1 h at room temperature (or overnight at 4 °C) with primary antibodies and 1 h at room temperature with secondary antibodies. Samples were then washed with PBS and mounted onto glass slides using Mowiol.

### Apoptosis assay

HeLa cells seeded in 384-well plates were infected with CTL2 as described in the infectious progeny assay. Cells were treated with Nutlin3a at the indicated concentrations from 12 h p.i. Ten hours post Nutlin3a treatment, medium was exchanged and recombinant human TNF-α (BD Pharmingen, 554618) was added at the indicated concentrations with 10 μg ml^−1^ cycloheximide (Sigma, 065K12261) together with Nutlin3a and incubated for 5 h until fixation and immunolabelling.

## Author contributions

E.G., M.R. and M.C.K. contributed equally. E.G. and M.R. conducted the western immunoblotting for Figs 1a, 2a,b and 4d and Supplementary Figs 1,2,3 and 5, the infectious progeny assay presented in Fig. 3a, the *Simkania* western immunoblotting and immunofluorescence presented in Fig. 4d and Supplementary Fig. 5, respectively, the apoptosis assay presented in Fig. 4e,f and Supplementary Fig. 6. M.C.K. conducted the western immunoblotting for Fig. 1e, the live imaging presented in Figs 1b–d and 2c and Supplementary Movies 1 to 5, conducted the confocal imaging presented in Fig. 4a and contributed to the analysis presented in Fig. 4b, Supplementary Fig. 4 and Supplementary Fig. 6b. M.A.Z and M.A. prepared the immunofluorescence samples and conducted the western immunoblotting for Fig. 4a,c and d and contributed to the analysis presented in Supplementary Fig. 6b. M.K. contributed to the analysis presented in Fig. 4b. V.B. conducted TEM and quantification presented in Fig. 3b,c and Supplementary Fig. 3. A.L. and T.F.M. triggered initiation of this study and A.L. provided the cells used for the live imaging and assisted in the analysis of the imaging experiments. T.F.M. supervised the project and the manuscript preparation.

## Additional information

**How to cite this article:** González, E. *et al.* *Chlamydia* infection depends on a functional MDM2-p53 axis. *Nat. Commun.* 5:5201 doi: 10.1038/ncomms6201 (2014).

## Supplementary Material

Supplementary Information Supplementary Figures 1-7

Supplementary Movie 1 Time-lapse videomicroscopy was performed from 24 h p.i. with CTL2 (MOI 0.5) using MCF7 p53-Venus reporter cells expressing pMT-p53-Venus and pEF1α-mCherry-53BP1. Infected cells are denoted by the expanding inclusions evident in brightfield. The interval between frames captured was 20 min and the duration is as indicated. Playback speed is 10 frames per second.

Supplementary Movie 2 Time-lapse videomicroscopy was performed from 24 h p.i. with CTL2 (MOI 0.5) using MCF7 p53-Venus reporter cells expressing pMT-p53-Venus and pEF1α-mCherry-53BP1 imaged in the presence of 5 μ Nutlin3a. Infected cells are denoted by the expanding inclusions evident in brightfield. The interval between frames captured was 20 min and the duration is as indicated. Playback speed is 10 frames per second.

Supplementary Movie 3 Time-lapse videomicroscopy was performed from 24 h p.i. with CTL2 (MOI 0.5) using MCF7 p53-Venus reporter cells expressing pMT-p53-Venus and pEF1α-mCherry-53BP1 imaged in the presence of 10 μg/ml chloramphenicol. Infected cells are denoted by the inclusions evident in brightfield. The failure of the inclusions to expand after addition of chloramphenicol highlights the efficacy of this bacteriostatic antimicrobial. The interval between frames captured was 20 min and the duration is as indicated. Playback speed is 10 frames per second.

Supplementary Movie 4 Time-lapse videomicroscopy was performed from 48 h p.i. with CTL2 (MOI 0.5) using MCF7 p53-Venus reporter cells expressing pMT-p53-Venus and pEF1α-mCherry-53BP1 imaged in the presence of 10 μg/ml chloramphenicol added 24 h p.i. Infected cells are denoted by the inclusions evident in brightfield. The failure of the inclusions to expand after addition of chloramphenicol highlights the efficacy of this bacteriostatic antimicrobial. The interval between frames captured was 20 min and the duration is as indicated. Playback speed is 10 frames per second.

Supplementary Movie 5 Time-lapse videomicroscopy was performed from 24 h p.i. with CTL2 (MOI 0.5) using MCF7 p53-Venus reporter cells expressing pMT-p53-Venus and pEF1α-mCherry-53BP1 imaged in the presence of 10 μM/ml CPAF-inhibitor peptide. Infected cells are denoted by the inclusions evident in brightfield. As reported previously, application of this peptide led to rapid induction of non-apoptotic cell death in CTL2-infected cells, severely attenuating the duration videomicroscopy could be performed. The interval between frames captured was 20 min and the duration is as indicated. Playback speed is 10 frames per second.
