# Supplementary material for: Chlamydia infection depends on a functional MDM2-p53 axis
Source: Nat Commun. 2014 Nov 13;5:5201. doi: 10.1038/ncomms6201 (PMC4243245; doi:10.1038/ncomms6201)
Supplement: Supplementary Information — Supplementary Figures 1-7 [file ncomms6201-s1.pdf]

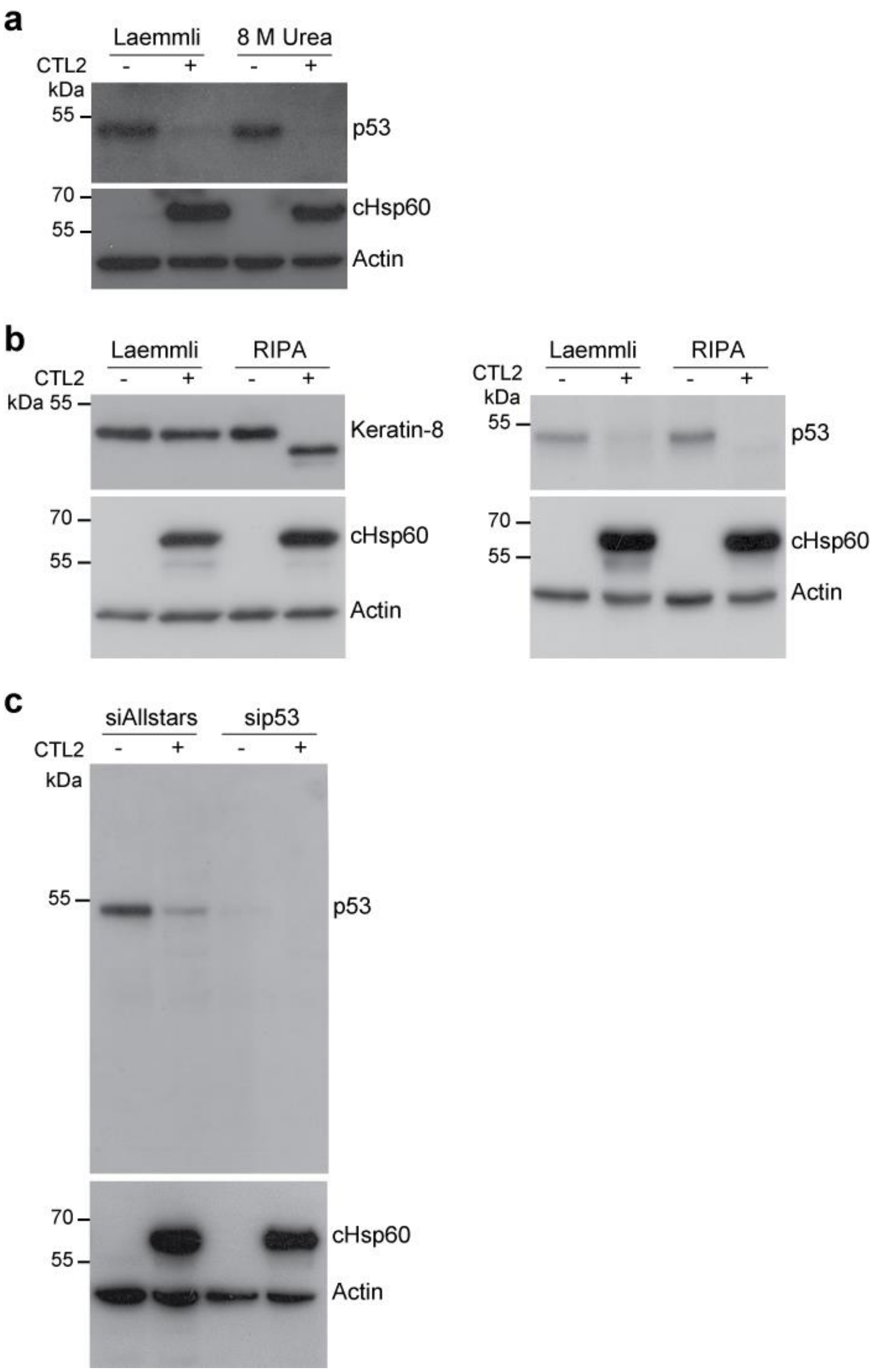

Supplementary Figure 1 | P53 is degraded following *Chlamydia* infection independent of the cell lysis and protein sample preparation procedure applied. (**a**) Western blotting analysis showing degradation of total p53 protein in CTL2-infected (MOI 1) whole cell lysates collected in Laemmli buffer or 8 M urea 48 h p.i. as indicated. Chlamydial Hsp60 and β-actin served as infection and loading controls, respectively. (**b**) Western blotting of whole cell lysates of HeLa cells infected with CTL2 (MOI 1) for 48 h and collected in Laemmli or RIPA lysis buffer. RIPA buffer lysates showed cleavage of keratin-8 while Laemmli buffer lysates demonstrated absence of post-lysis proteolytic effects as monitored by the absence of keratin-8 cleavage. Importantly, CTL2-infected protein samples lysed in Laemmli buffer maintained reduction in p53 levels, thus further demonstrating that p53 degradation occurs prior to cell lysis. Chlamydial Hsp60 and β-actin served as infection and loading controls, respectively. (**c**) Western blotting of whole cell lysates extracted from HeLa cells treated with control RNAi oilgonucleotides (siAllstars) or those specifically targeted against p53 and then infected with CTL2 (MOI 1) for 48 h confirm that the degraded protein is p53. Chlamydial Hsp60 and β-actin served as infection and loading controls, respectively.

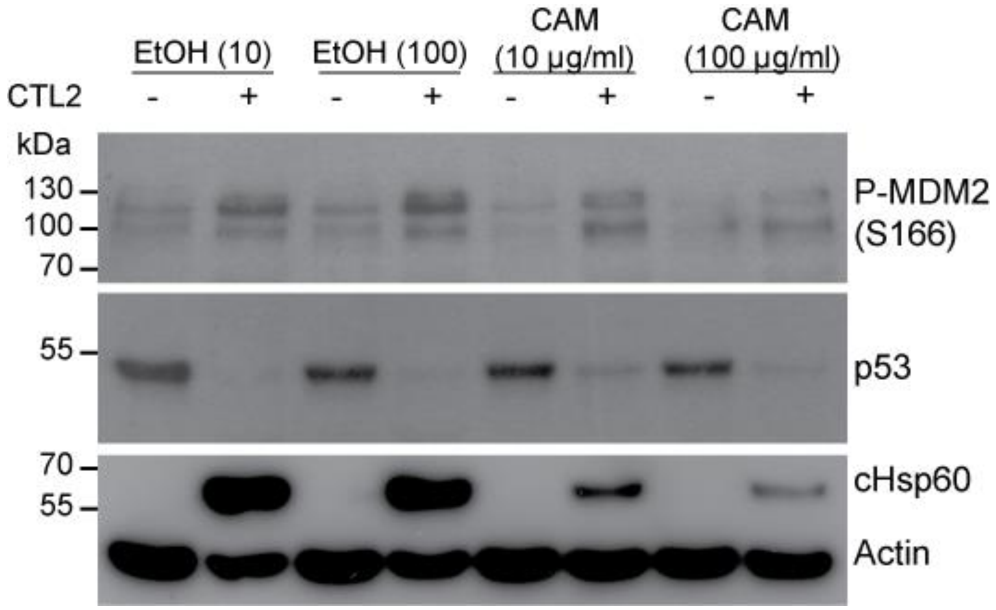

Supplementary Figure 2 | Inhibition of chlamydial growth with chloramphenicol from 24 h p.i. does not rescue p53 degradation in *Chlamydia*-infected cells. Western blotting analysis showing degradation of total p53 protein and an increase in P-MDM2 (S166) in CTL2-infected whole cell lysates (MOI 1) treated with increasing concentrations of chloramphenicol (CAM) from 24 h p.i. as indicated. Protein samples were collected 48 h p.i. Chlamydial Hsp60 and β-actin served as infection and loading controls, respectively.

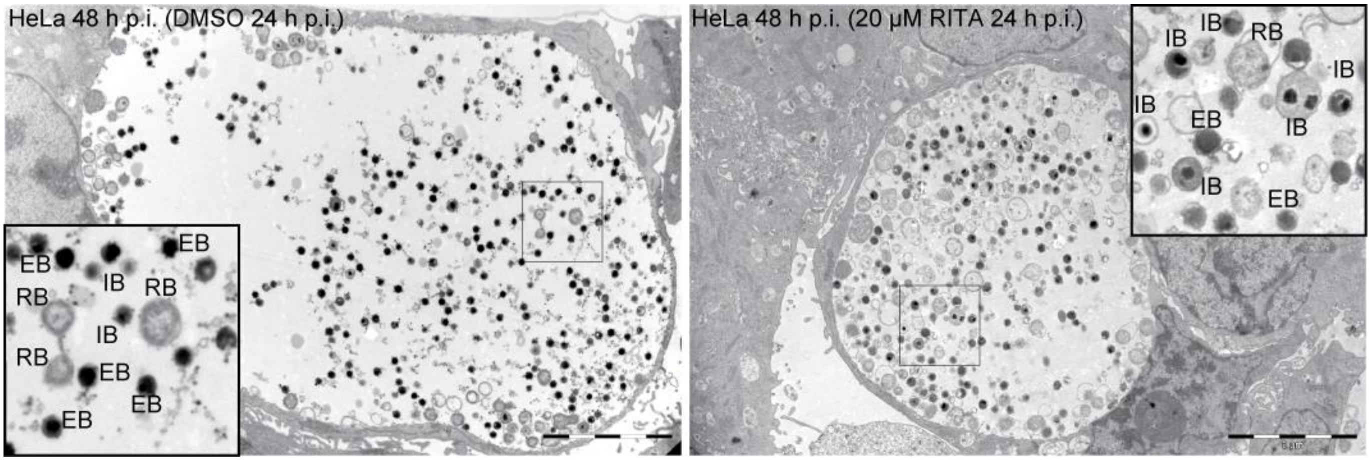

Supplementary Figure 3 | Inhibition of the MDM2-p53 interaction with RITA decreases the proportion of EBs. Representative transmission electron micrographs of infected cells 48 h p.i., with or without RITA from 24 h p.i. Scale bars: 5 µm. Insets present high resolution images of the indicated region. Similar to Nutlin3a treatment (Fig. 3b), RITA treatment significantly decreased the proportion of EBs, while the proportion of RBs and IBs increased.

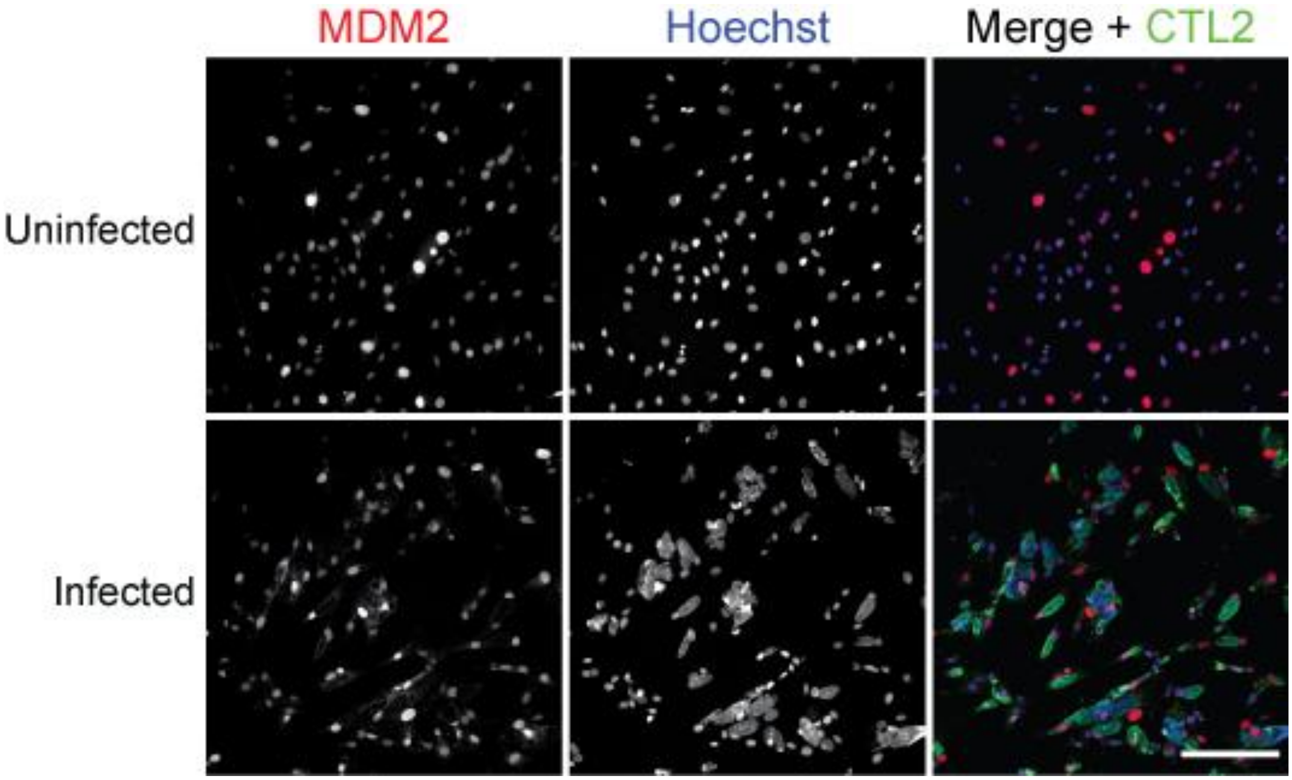

Supplementary Figure 4 | Total cellular MDM2 levels are not influenced by *Chlamydia* infection. CTL2 infection does not reduce the number of MDM2 positive nuclei in fallopian tube primary mesenchymal cell monolayers. *Chlamydia* inclusions and nuclei were labelled with a *Chlamydia* antibody and Hoechst. Scale bar: 200 µm.

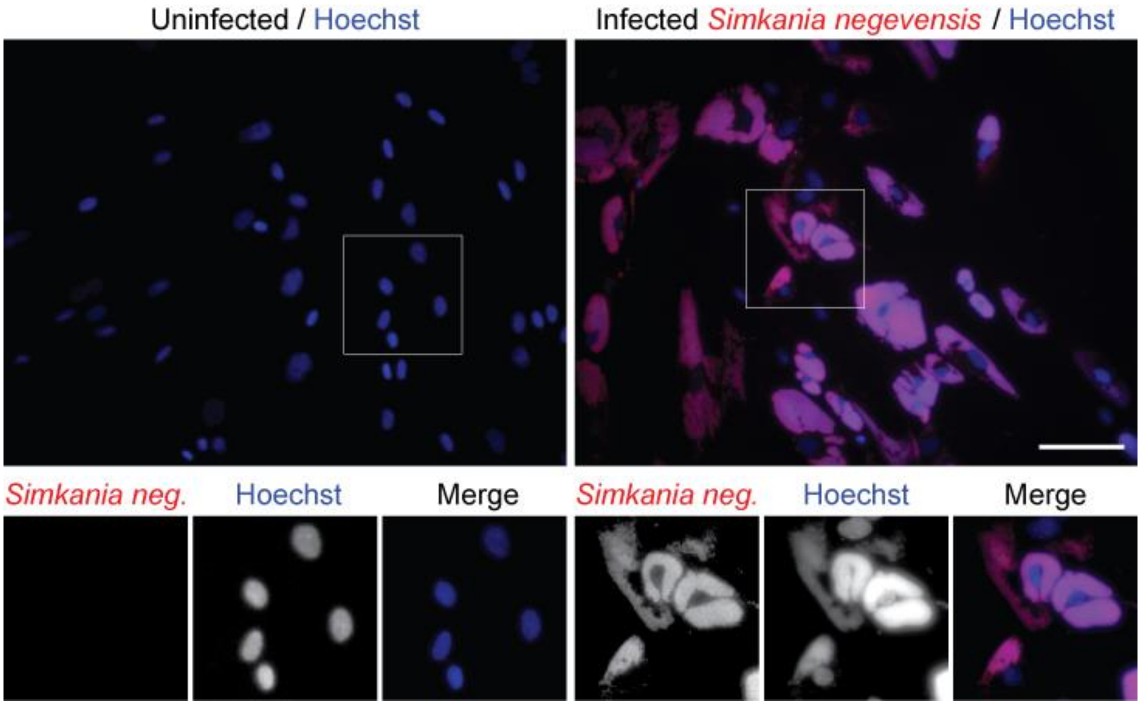

Supplementary Figure 5 | *Simkania negevensis* infection of primary cells. Immunofluorescent labelling of uninfected and infected primary mesenchymal cells (MOI 2, 72 h p.i.) using anti-SN-GroEL antibody, demonstrating extensive infection with this intracellular bacterium. Hoechst was used for nuclear staining. Scale bar: 200 µm.

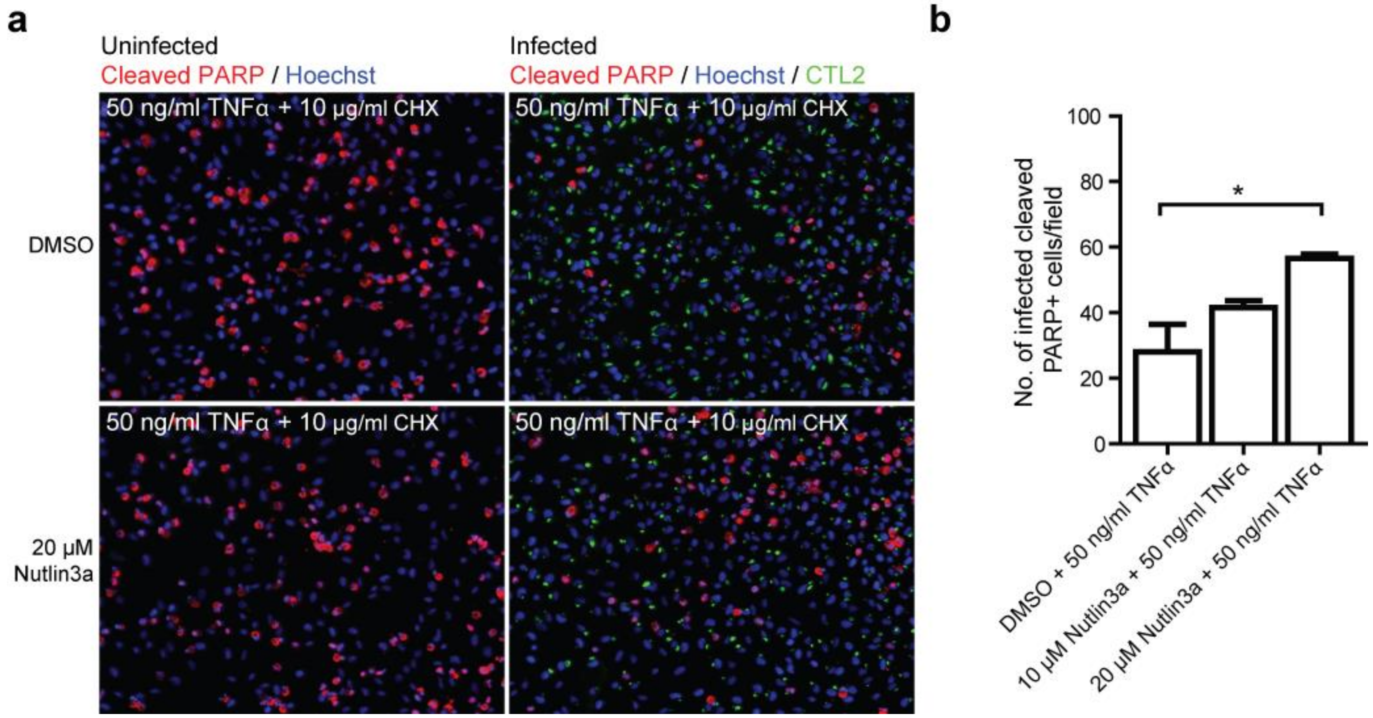

Supplementary Figure 6 | *Chlamydia*-induced resistance to apoptosis is partially rescued following treatment with Nutlin3a. (**a**)The extent of apoptosis induced by TNFα treatment was analysed with cleaved PARP antibodies in HeLa cells infected with CTL2 (MOI 1). Infected cells showed greatly reduced labelling for cleaved PARP, however, Nutlin3a treatment partially restored the number of cleaved PARP-positive cells. *Chlamydia* inclusions and nuclei were labelled with anti-*Chlamydia* antibody and Hoechst. (**b**) Number of cleaved PARP-positive nuclei per image in infected HeLa cells treated with Nutlin3a during apoptosis induction with TNFα and cycloheximide, shows that Nutlin3a significantly increases the number of apoptotic nuclei in infected populations of cells. Results are depicted as mean ± SD of two independent experiments; * $p < 0.05$, one-way ANOVA with Bonferroni post-hoc test.

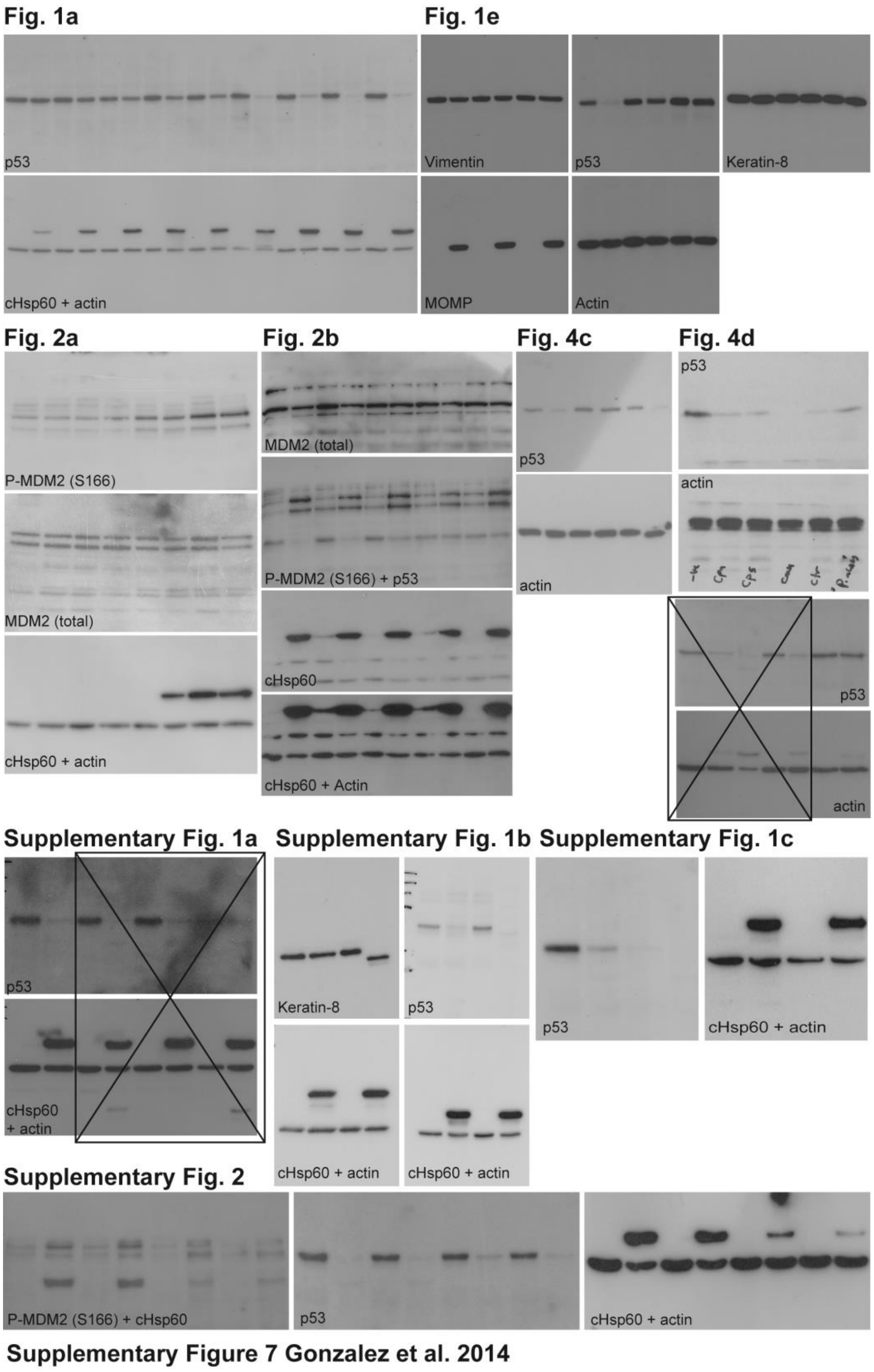

Supplementary Figure 7 | Full scans of all blots presented in cropped format throughout the manuscript.
